# Clustering-Based Identification of BMI-Associated Metabolites with Mechanistic Insights from Network Analysis in Korean Men

**DOI:** 10.3390/metabo15020088

**Published:** 2025-02-02

**Authors:** JooYong Park, Jihyun Kang, Ji-Yeoun Lee, Daehee Kang, Joo-Youn Cho, Ji-Yeob Choi

**Affiliations:** 1 Department of Big Data Medical Convergence, Eulji University, Seongnam-si 13135, Republic of Korea; judepark0501@gmail.com (J.P.); jylee@eulji.ac.kr (J.-Y.L.); 2 Department of Biomedical Sciences, Seoul National University Graduate School, Seoul 03080, Republic of Korea; dhkang@snu.ac.kr; 3 Department of Clinical Pharmacology and Therapeutics, College of Medicine and Hospital, Seoul National University, Seoul 03080, Republic of Korea; jikang@snu.ac.kr; 4 Department of Preventive Medicine, College of Medicine, Seoul National University, Seoul 03080, Republic of Korea; 5 Cancer Research Institute, Seoul National University, Seoul 03080, Republic of Korea

**Keywords:** targeted metabolomics, modifiable behavior, clustering, network analysis

## Abstract

Background: Epidemiological studies using metabolomics often encounter challenges due to metabolite profiles being influenced by multiple modifiable behavioral factors, including regular exercise, smoking, drinking, and weight control. This study aimed to identify modifiable behavioral factors reflected in metabolites by clustering subjects based on their metabolite profiles. Networks of metabolites were constructed to visualize their relationships and the differences between clustering groups. Methods: Sixty-four healthy men were included in this study. Information on regular exercise, smoking, and drinking was collected by questionnaires, and body mass index (BMI), an indicator of weight control, was calculated based on measured height and weight. Through targeted metabolomics, the concentrations of 149 metabolites were quantified. Subjects were clustered using the k-means method based on metabolite composition. Correlation-based networks were constructed for each cluster using Cytoscape software, followed by network analysis. Results: The subjects were divided into two clusters, with BMI identified as a distinguishing feature. Four lyso-phosphatidylcholines (PCs), six diacyl-PCs, and one acyl-alkyl-PC were positively associated with BMI. In the constructed network, acyl-alkyl-PCs exhibited the highest degrees, suggesting their central role in BMI-associated metabolic pathways. Conclusions: These findings suggest that metabolites can reflect behavioral factors, with BMI exerting a significant influence on metabolite profiles, particularly through its associations with phosphatidylcholines.

## 1. Introduction

Physical inactivity, smoking and drinking habits, and obesity are well known as modifiable behavioral factors. The reported leading risk factors for global mortality in 2021 included high body mass index (BMI) (5th for female and 6th for male), smoking (tobacco use) (6th for female and 1st for male), alcohol use (14th for female and 8th for male) and physical inactivity (13th for female and 15th for male) [1]. Moreover, they are associated with various chronic diseases and make a large contribution to the burden of diseases globally [2,3,4,5]. Despite these associations, the combined and complex physiological mechanisms through which modifiable behaviors influence health outcomes remain poorly understood.

Among various biomarkers, metabolites are more closely related to phenotypes than genes or proteins because metabolites are biochemical end-products [6]. Metabolites reflect biological pathways such as enzyme activities and provide a snapshot of metabolic states or diseases [6,7,8,9]. Previous studies have shown associations between each modifiable behavior and metabolites with a hypothetical approach. For example, physical activity was positively associated with amino acids, coffee intake was inversely associated with phospholipids, and smoking was positively associated with acylcarnitines [7,8]. Alcohol consumption has been linked to various metabolites, including threonine, guanidinosuccinate, and glutamine, which are associated with liver enzyme activity [10]. BMI has also been significantly associated with multiple metabolites, including increased levels of aromatic and branched-chain amino acids (e.g., tyrosine, valine, isoleucine, phenylalanine) and decreased levels of glycerophospholipids, such as lysophosphatidylcholines and acyl-alkyl-phosphatidylcholines [11]. However, metabolites are unlikely to be influenced by a single factor; rather, multiple factors might be reflected in the metabolite status. Individuals with similar behaviors probably have similar metabolite profiles. Therefore, clustering methods in the context of the exploratory approach that divide subjects into optimal groups according to their similarities or dissimilarities could help identify shared or distinguishing modifiable factors reflected in metabolite status within or between cluster groups [12,13,14]. In addition, network analysis, which illustrates interactions between metabolites, could provide insights into the biological mechanisms linking behaviors to health outcomes [15].

We aimed to examine which modifiable behaviors, including physical activity, smoking, drinking habits, and BMI (an indicator of weight control) are reflected in metabolites by clustering the subjects according to their metabolite compositions and comparing the difference between clustering groups. Moreover, networks of metabolites were used to show the relationships between metabolites, the differences between clustering groups, and associations with a distinguishing feature.

## 2. Methods

### 2.1. Study Population

Healthy adults aged 20 to 69 were recruited from local health examination centers between 2010 and 2012 as a control pool in a previous case–control study [16]. The original dataset consisted of 53,495 subjects, all of whom were interviewed face-to-face by trained interviewers. Sixty-four subjects were randomly selected from those who had no missing information on key modifiable behavioral factors (regular exercise, BMI, smoking, and alcohol consumption habits) and who also provided a blood sample. The sample size of 64 was determined based on a priori power analysis using G*Power software (ver. 3.1.9.7), which calculated the required sample size specifically for linear multiple regression analysis. The calculation assumed an effect size (f^2^) of 0.25 (a medium value between 0.15 and 0.35), a significance level (α) of 0.05, a statistical power (1 − β) of 0.85, and 5 predictors. These parameters yielded a required sample size of 64, ensuring sufficient power for the study objectives [17]. Selecting 64 subjects ensured statistical robustness while avoiding the unnecessary use of resources. This focused sampling approach also accounted for the high-quality data required for metabolomic analysis, as only individuals with complete data were included, minimizing potential biases. Additionally, given the exploratory nature of this study, a smaller, well-defined subset was appropriate for efficiently generating hypotheses regarding the relationship between modifiable behaviors and metabolite profiles. All participants signed consent forms. The study was approved by the Committee on Human Research of Seoul National University Bundang Hospital (IRB No. B-1004/097-014). This study was performed in accordance with the Declaration of Helsinki.

### 2.2. Modifiable Behavioral Factors

Information on participation in regular exercise, smoking, and alcohol consumption habits was collected by questionnaire. The question was “Do you exercise regularly enough to sweat?”, and the subjects answered yes or no. The subjects who participated in regular exercise answered further questions regarding frequency per week and average duration. Smoking status was categorized as never, former and current. The drinking habit questionnaire was categorized into yes or no. BMI as an indicator of weight control was calculated by measured height and weight (kg/m^2^). Obesity was defined when BMI > 25 kg/m^2^, following the 2018 Korean Society for the Study of Obesity Guideline [18].

### 2.3. Measurement of Serum Metabolites Concentration

Blood samples were collected from subjects after they fasted for at least 8 h. Serum samples were obtained by centrifugation (3000 rpm at 4 °C for 10 min) and were stored in a freezer at −80 °C until analysis. All serum samples were processed using the AbsoluteIDQ p180 kit (BIOCRATES Life Sciences AG, Innsbruck, Austria) with liquid chromatography mass spectrometry (LC-MS/MS). Mass spectrometric analysis was performed on an API 4000 QTRAP (Applied Biosystems/MDS Sciex, Foster City, CA, USA) equipped with an Agilent 1200 series high-performance liquid chromatography (HPLC) system (Agilent Technologies, Santa Clara, CA, USA). The AbsoluteIDQ p180 kit assay combines flow injection analysis (FIA) and liquid chromatography (LC), which can quantify 188 metabolites from five classes—amino acids, biogenic amines, glycerophospholipids, sphingomyelins, acylcarnitines, and hexose. Metabolites were quantified and quality assessments were performed using MetIDQ software (Biocrates). Ten microliters of serum samples were added to a 96-well extraction plate, which contained the internal standards and were dried under nitrogen gas. After derivatization with phenyl isothiocyanate in ethanol/water/pyridine (ratio 1/1/1, *v*/*v*/*v*), metabolites and internal standards were extracted with 5 mM ammonium acetate in methanol for LC-MS/MS and FIA analyses. After excluding metabolites that measured below the limit of detection (LOD) for more than 10% of participants, we used 149 metabolites, including 21 amino acids, 18 acylcarnitines, 84 phosphatidylcholines (PCs) (12 lyso-, 36 diacyl-, 36 acyl-alkyl-), 12 biogenic amines, 13 sphingomyelins, and 1 hexose, in this study. The remaining measurements below the LOD were imputed to half the LOD value of each metabolite.

### 2.4. Statistical Analysis

All analyses were performed in R software (ver. 4.0.0). To normalize metabolite concentrations, we employed normal score transformation using the “gstat” package in R to generate a normal distribution and to unify scales. This method was chosen because metabolite concentrations vary significantly in scale, and some metabolites exhibit non-normal distributions. Normal score transformation ensures fair comparisons by standardizing metabolite data (mean = 0, standard deviation = 1) and minimizing the influence of outliers. The Euclidean distance matrix was obtained through the function “vegdist” in the “vegan” package. Hierarchical clustering based on Euclidean distance and the Ward method was performed by the function “hclust” in the “stats” package [12,19]. The optimal number of clusters was decided from the largest average silhouette width by the function “silhouette” in the “cluster” package. The negative value of the silhouette width, which was obtained from the function “silhouette” in the “cluster” package, was used to determine misclassification. The function “kmeans” in the “stats” package was used with the predetermined number of groups to perform k-means clustering [12].

The difference between clustering groups was tested by the t-test for continuous variables and by the chi-square test for categorical variables. General linear models were used to examine the associations between metabolites and modifiable factors. The dependent variables were metabolites, and the independent variables were age, BMI, regular exercise, smoking, and drinking habits. Multiple comparisons were adjusted by the false discovery rate (FDR) [20].

Pearson correlation coefficient matrices were obtained from each cluster using R software (ver. 4.0.0), specifically utilizing the corr.test function from the “psych” package. Networks were constructed in clusters 1 and 2 separately, among correlation coefficients that were over the threshold (|r| ≥ 0.5 and *p* < 0.001) in Cytoscape software (ver. 3.7.2). A rather strict threshold was used to visualize more informative and lucid topology. Positive correlations have been visualized as solid edges, and negative correlations as dotted edges. The widths of edges represent the magnitudes of correlation coefficients. Circle nodes indicate metabolites associated with BMI, which was the only significant variable associated with the clustering groups after the general linear model, as described above. “Analyze Network” was performed in Cytoscape software (ver. 3.7.2) to calculate the “degree” (the number of connected edges), which suggests a hub node that plays a central role in the relationships, and the “betweenness centrality” (the average number of shortest paths that go through the node), which implies the key node that plays an important role in the network [21].

## 3. Results

A dendrogram was obtained from the hierarchical clustering of the chord distance matrix based on metabolite data (Figure S1), and the optimum number of cluster groups was two, which was the largest average silhouette width (Figure S2). Cluster 1 included 50 men, and cluster 2 included 14 men; however, the misclassification was shown by the silhouette plot (Figure S3). When the subjects were clustered by k-means partitioning with two groups, we observed no misclassification, and these cluster groups (41 men in cluster 1 and 23 men in cluster 2) were used in this study (Figure S4).

**Figure 1:**
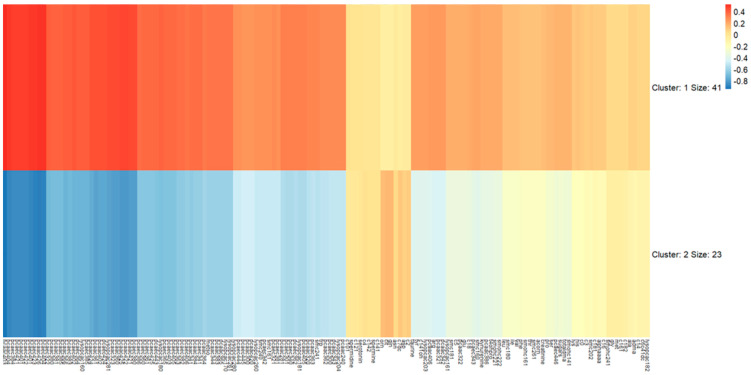
Heatmap of metabolite profiles by two clustering groups (color represents normalized metabolite intensities).

The basic characteristics of the subjects and the differences between the clustering groups are shown in Table 1. The mean age of the subjects was 40.2 years, and 34.4% of the men were obese. More than half of the subjects participated in regular exercise (60.9%). Approximately one-third of the men were current smokers, and most of the men were current drinkers. Age and modifiable behavioral factors were not different between clusters 1 and 2. However, BMI was higher in cluster 1 than in cluster 2 (Table 1), and BMI was significantly associated with cluster groups (OR = 0.77, 95% CIs: 0.61–0.98) (Table 2). The heatmap illustrates the normalized metabolite intensities, clearly differentiating the two clusters (Figure 1). Cluster 1 (*N* = 41) showed distinct metabolic patterns compared to cluster 2 (*N* = 23), with marked differences observed in phosphatidylcholines and other lipid-related metabolites.

Among 149 metabolites, BMI was positively associated with 11 metabolites, including four lyso-PCs, six diacyl-PCs and one acyl-alkyl PC, after adjusting for multiple comparisons (FDR-*p* < 0.05) (Table 3).

**Figure 2:**
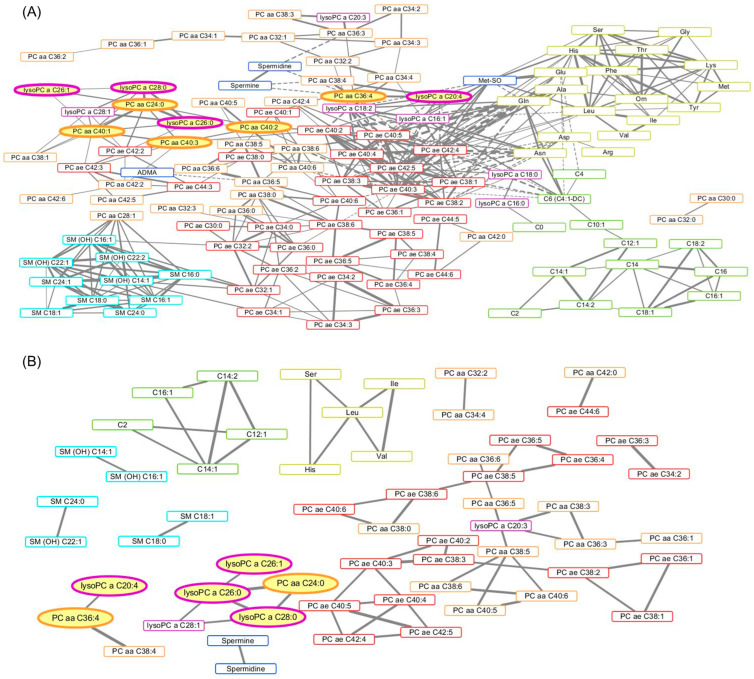
Correlation-based network of metabolites and their associations with BMI. The network in cluster 1 consisted of 122 nodes and 405 edges (**A**), and the network in cluster 2 consisted of 58 nodes and 61 edges (**B**). Threshold: |r| ≥ 0.4 and *p* < 0.001. Circle nodes: metabolites significantly associated with BMI. Solid edges: positive correlations. Dotted edges: negative correlations. Width of edges: magnitudes of correlation coefficients. Border color: red = diacyl-phosphatidylcholines, orange = acyl-alkyl-phosphatidylcholines, purple = lyso-phosphatidylcholines, blue = biogenic amines, cyan = sphingomyelins, green = acylcarnitines, yellow = amino acids. Abbreviations: lysoPC = lyso-phosphatidylcholine; a = acyl; aa = diacyl; ae = acyl-alkyl; PC= phosphatidylcholine; SM = sphingomyelin; C = acylcarnitine. Networks were visualized by Cytoscape software (ver.3.7.2).

In cluster 1, 1267 pairs of metabolites were correlated (*p* < 0.05), while 305 pairs of metabolites were correlated in cluster 2 (*p* < 0.05). Correlation networks of metabolites were produced for clusters 1 and 2 separately among correlation coefficients that were over the threshold (|r| ≥ 0.5 and *p* < 0.001) (Figure 2). More nodes and edges were observed in cluster 1 (122 nodes and 405 edges) than in cluster 2 (58 nodes and 61 edges). Among the 11 metabolites that were associated with BMI, all four lyso-PCs were included in the networks of both clusters 1 and 2. Five associated diacyl-PCs were observed in the network of cluster 1, while only two associated diacyl-PCs were found in the network of cluster 2.

However, lyso-PCs and diacyl-PCs were not placed at the center of the networks, as they did not show high degree or betweenness centrality according to the network analysis (Table 4). Acyl-alkyl-PCs (PC ae C40:3 and PC ae C42:5) showed high degree and betweenness centrality in the network of cluster 1. In the network of cluster 2, leucine showed the highest degree and betweenness centrality (Table 4).

## 4. Discussion

In this study, the healthy men were clustered into two groups based on their metabolite profiles. BMI emerged as a distinguishing characteristic, and we found that four lyso-phosphatidylcholines (lyso-PCs), six diacyl-phosphatidylcholines (diacyl-PCs), and one acyl-alkyl-phosphatidylcholine (acyl-alkyl-PC) were positively associated with BMI. Network analysis revealed that acyl-alkyl-PCs (specifically PC ae C42:5 and PC ae C40:3) exhibited higher degrees of connectivity compared to lyso-PCs and diacyl-PCs, suggesting a potentially central role for acyl-alkyl-PCs in BMI-associated metabolic and endocrine pathways.

The associations between lyso-PCs and BMI were inconsistent with previous studies. In the present study, four lyso-PCs (lysoPC a C20:4, lysoPC a C26:0, lysoPC a C26:1, and lysoPC a C28:0) showed positive associations with BMI, while a few studies found inverse associations between lyso-PCs and BMI [8,22,23,24,25]. In particular, lysoPC a C18:1 and C18:2 were commonly found in those studies, and both also showed inverse associations with waist circumference [26]. However, lysoPC a C28:0 was positively associated with BMI in the EPIC-Oxford study [23], and a twin study also reported that lyso-PCs were higher in the obese subjects [27]. In the mouse experiment, lyso-PCs that had fewer than 20 carbons decreased when following the high-fat diet’s time course, whereas lysoPC a C20:4 was elevated [28]. Therefore, these inconsistent results imply that the associations between lyso-PCs and BMI depend on the number of carbon atoms and possibly the number of double bonds due to the differences in their biological properties [23,29]. Further studies are needed to examine the biological differences according to the chain length and the degree of saturation.

The positive associations between diacyl-PCs and BMI are consistent with the findings of prior studies [8,21,22,23]. Among six diacyl PCs associated with BMI in our results, PC aa C36:4, PC aa C40:3, and PC aa C40:4 were also found in the EPIC-Oxford study, and they were positively associated with BMI, although only PC aa C40:4 was significant [23]. These associations may reflect underlying mitochondrial dysfunction, a hallmark of obesity-related metabolic disorders. Obesity-induced mitochondrial dysfunction could enhance oxidative stress and reduce fatty acid oxidation. Such mitochondrial changes could be influenced by altered endocrine regulation, particularly through insulin resistance and chronic inflammation. Moreover, obesity could influence the enzymes that are related to phospholipid regulation. These mechanisms could lead to elevated levels of phosphatidylcholines in blood [24,30,31,32].

A strict threshold was applied in the network analysis to ensure the resulting topology was both informative and visually clear. Relaxing the threshold to *p* < 0.01 still resulted in all absolute correlation coefficients remaining above 0.5, but increased the number of edges in the cluster 1 network to 747—nearly double the current count—making interpretation inefficient and visually cumbersome. To balance clarity and rigor, the threshold of *p* < 0.001 was chosen, focusing on stronger correlations that remain robust under stringent criteria. This approach enhances the reliability and interpretability of the mechanistic insights derived from the analysis. In this context, an acyl-alkyl-PC (PC ae C42:1) was not presented in the networks due to the threshold (|r| ≥ 0.5 and *p* < 0.001), although it was associated with BMI. It was actually correlated with 10 metabolites (*p* < 0.05), and 1 of them was PC aa C40:4 (r = 0.5379, *p* = 0.0094, data not shown), which was also not visualized in the network. This exclusion highlights the trade-off between visual clarity and the inclusion of potentially relevant but less statistically robust relationships. Nevertheless, the acyl-alkyl-PCs (PC ae C42:5, PC ae C40:3) had the greatest number of edges (the highest degree number) in the network of cluster 1, which suggests that the acyl-alkyl-PCs are more likely to play central roles among them. Among the metabolites associated with BMI, the majority were lyso-PCs and diacyl-PCs, while only one acyl-alkyl-PC (PC ae C42:1) showed a significant association. A previous study found elevated levels of acyl-alkyl-PCs in individuals with higher waist circumference [26], while another study showed that some acyl-alkyl-PCs were negatively associated with obesity, and others were positively associated [33]. The positive association between PC ae C42:1 and BMI may be attributed to obesity-induced metabolic alterations, such as disrupted fatty acid metabolism and oxidative stress, influenced by the structural characteristics of acyl-alkyl-PCs [34]. The mechanisms of the acyl-alkyl-PC in the relationships of metabolites remain to be elucidated, although previous studies have suggested interrelationships between phosphatidylcholines, metabolisms, and transfer pathways [8,35,36]. Moreover, the network of cluster 1 had more nodes and edges than the network of cluster 2 in the same threshold (Figure 1), indicating that the relationships or interactions between metabolites are stronger in subjects in cluster 1, who have relatively higher BMIs. Indeed, clusters 1 and 2 were classified by metabolic status, meaning that both groups include obese men according to BMI criteria; there is only a difference in proportion. As shown in a previous study that found outliers such as obese individuals having healthy metabolomes and the normal individuals having obese metabolomes [37], obese men in cluster 2 could have relatively healthy metabolite profiles, and the difference in clinical status of obesity might be reflected in the networks.

We acknowledge several limitations in this study. First, the cross-sectional design precludes the inference of a causal relation between BMI and metabolites. However, BMI, as an indicator of weight control, represents a characteristic shaped by prior behaviors and lifestyle choices. Therefore, the BMI measured at study enrollment likely reflects weight control behaviors preceding enrollment, and consequently could have influenced the metabolite profile observed at the time of blood collection. Second, the sample size here was smaller than in previous studies. In addition, there was a lack of information regarding modifiable behavioral factors such as the amount of alcohol intake, smoking pack–years, or dietary habits. Further studies with more diverse information and a replication study with a larger study sample size would be needed to infer robust features and suggest mechanisms in depth. Nevertheless, we derived significant results regarding the relation between BMI and a few metabolites after adjusting multiple comparisons (FDR-*p* < 0.05). Lastly, the results from the present study cannot be generalized because we used only men as the study subjects. Given that there are many differences in basic characteristics between men and women, especially when considering the effects of hormones, this limitation is particularly important. However, conducting the study in men, who are less influenced by hormonal fluctuations compared to women, may have reduced potential confounding effects related to hormonal variability, especially in metabolomics research. Moreover, differences in metabolic profiles between sexes were also reported in a previous study [38]. Consequently, sex-specific studies are needed to further establish evidence of metabolic differences and sex-specific characteristics.

## 5. Conclusions

Metabolites could reflect multiple behavioral factors related to the health of individuals. In this study, healthy men were clustered into two groups according to their metabolite profiles, and we found that BMI, as an indicator of weight control, was a key factor distinguishing the two clusters. Thereby, only phosphatidylcholines (a class of lipid metabolites) emerged as notable factors, highlighting their potential role in metabolic pathways influenced by BMI. The network analysis further suggests that acyl-alkyl-phosphatidylcholines may play a central role in the metabolic mechanisms underlying obesity-prone groups. If data on additional metabolites become available following the development of metabolomics, it would help to elucidate the biological and physiological mechanisms linking BMI, metabolite profiles, and overall health.

## Figures and Tables

**Table 1:** Characteristics of the study population by clusters.

	Total		Cluster 1 (*N* = 41, 64.1%)		Cluster 2 (*N* = 23, 35.9%)		
	*N*	(%)	*N*	(%)	*N*	(%)	*p*-Value
Age (year), mean ± SD	40.2 ± 7.01		40.0 ± 6.25		40.6 ± 8.33		0.7696 ^a^
<40	29	(45.3)	20	(48.8)	9	(39.1)	0.6478 ^b^
40–50	29	(45.3)	18	(43.9)	11	(47.8)	
≥50	6	(9.4)	3	(7.3)	3	(13.0)	
BMI (kg/m^2^), mean ± SD	24.1 ± 2.62		24.7 ± 2.56		23.0 ± 2.46		0.0152 ^a^
<25	40	(62.5)	23	(56.1)	19	(82.6)	0.0321 ^b^
≥25	22	(34.4)	18	(43.9)	4	(17.4)	
Regular exercise							
No	25	(39.1)	15	(36.6)	10	(43.5)	0.5876 ^b^
Yes	39	(60.9)	26	(63.4)	13	(56.5)	
Smoking							
Never	17	(26.6)	11	(26.8)	6	(26.1)	0.8932 ^b^
Former	27	(42.2)	18	(43.9)	9	(39.1)	
Current	20	(31.3)	12	(29.3)	8	(34.8)	
Drinking							
No	5	(7.8)	3	(7.3)	2	(8.7)	0.8437 ^b^
Yes	59	(92.2)	38	(92.7)	21	(91.3)	

^a^ *t*-test. ^b^ chi-square test.

**Table 2:** Associations between characteristics of the study population and clusters.

	Cluster 2 vs. 1			
	Crude Model		Adjusted Model	
	OR	(95% CI)	OR ^a^	(95% CI)
Age	1.01	(0.94–1.09)	1.00	(0.92–1.09)
BMI	0.76	(0.60–0.96)	0.76	(0.60–0.96)
Regular exercise				
Yes	0.75	(0.27–2.12)	0.79	(0.26–2.40)
Smoking				
Former	0.92	(0.26–3.29)	0.97	(0.24–3.89)
Current	1.22	(0.32–4.66)	1.48	(0.35–6.30)
Drinking				
Yes	0.83	(0.13–5.36)	0.92	(0.10–8.20)

^a^ Logistic regression adjusting for age, BMI, regular exercise, smoking status, and alcohol habit.

**Table 3:** Metabolites associated with body mass index.

#	Metabolites	β-Coefficient	Standard Error	*p* Value	FDR-p	Bonferroni-p
1	lysoPC a C28:0	0.1741	0.0426	0.0001	0.0165	0.0209
2	lysoPC a C26:0	0.1711	0.0433	0.0002	0.0165	0.0330
3	PC aa C24:0	0.1585	0.0433	0.0006	0.0278	0.0835
4	lysoPC a C20:4	0.1507	0.0424	0.0008	0.0289	0.1157
5	PC aa C40:2	0.1480	0.0449	0.0017	0.0359	0.2514
6	PC aa C40:3	0.1383	0.0421	0.0018	0.0359	0.2658
7	PC ae C42:1	0.1475	0.0457	0.0021	0.0359	0.3091
8	PC aa C40:4	0.1429	0.0448	0.0023	0.0359	0.3454
9	lysoPC a C26:1	0.1457	0.0458	0.0024	0.0359	0.3571
10	PC aa C40:1	0.1404	0.0442	0.0024	0.0359	0.3591
11	PC aa C36:4	0.1321	0.0421	0.0027	0.0370	0.4074
12	PC aa C38:3	0.1365	0.0467	0.0050	0.0623	0.7472
13	lysoPC a C28:1	0.1304	0.0457	0.0061	0.0686	0.9112
14	PC aa C38:4	0.1215	0.0429	0.0064	0.0686	0.9598
15	lysoPC a C16:0	0.1299	0.0470	0.0077	0.0691	1.0000
16	lysoPC a C20:3	0.1283	0.0465	0.0078	0.0691	1.0000
17	PC ae C42:2	0.1217	0.0442	0.0079	0.0691	1.0000
18	PC aa C42:2	0.1137	0.0456	0.0156	0.1293	1.0000
19	PC aa C36:3	0.1129	0.0476	0.0213	0.1606	1.0000
20	PC aa C38:1	0.1080	0.0464	0.0234	0.1606	1.0000
21	Glutamate	0.1092	0.0473	0.0246	0.1606	1.0000
22	C5	0.1107	0.0482	0.0255	0.1606	1.0000
23	PC ae C30:1	0.1071	0.0468	0.0258	0.1606	1.0000
24	SM C24:0	0.1083	0.0473	0.0259	0.1606	1.0000
25	Hexose	0.0954	0.0456	0.0410	0.2399	1.0000
26	PC ae C38:4	0.0972	0.0467	0.0419	0.2399	1.0000
27	PC ae C40:2	0.0972	0.0477	0.0462	0.2499	1.0000

Note. Only metabolites with *p* < 0.05 are shown. Abbreviations: lysoPC = lyso-phosphatidylcholine; a = acyl; aa = diacyl; ae = acyl-alkyl; PC = phosphatidylcholine; SM = sphingomyelin; C = acylcarnitines. Adjusted for age, smoking, drinking, and participation in regular exercise.

**Table 4:** Results of the network analyses. (A) Network analysis in cluster 1 and the associations between metabolites and BMI. (B) Network analysis in cluster 2 and the associations between metabolites and BMI.

		(A) Parameters of the Network Analysis			Associations with BMI			
Rank	Metabolite	Degree	Betweenness Centrality	Closeness Centrality	β-Coefficient	Standard Error	*p* Value	FDR-p
1	PC ae C40:3	23	0.4960	0.3684	0.0746	0.0474	0.1210	0.3539
2	PC ae C42:5	23	0.1111	0.3352	0.0400	0.0472	0.4002	0.6855
3	PC ae C42:4	20	0.0690	0.3306	0.0446	0.0487	0.3636	0.6855
4	PC ae C40:5	17	0.0208	0.3083	0.0407	0.0473	0.3928	0.6855
5	Gln	17	0.0107	0.3156	−0.0413	0.0502	0.4136	0.6924
6	PC ae C40:2	16	0.0898	0.3140	0.0972	0.0477	0.0462	0.2499
7	PC ae C38:1	15	0.0391	0.3148	−0.0116	0.0469	0.8059	0.9455
8	Leu	14	0.0910	0.3005	0.0053	0.0496	0.9152	0.9869
9	His	14	0.0280	0.2668	0.0163	0.0503	0.7466	0.9027
10	PC ae C40:4	14	0.0018	0.3028	0.0928	0.0474	0.0550	0.2499
31	PC aa C24:0	9	0.0466	0.2338	0.1585	0.0433	0.0006	**0.0278**
32	PC aa C40:1	9	0.0071	0.2077	0.1404	0.0442	0.0024	**0.0359**
36	PC aa C36:4	8	0.0978	0.2680	0.1321	0.0421	0.0027	**0.0370**
41	lysoPC a C20:4	8	0.0110	0.2656	0.1507	0.0424	0.0008	**0.0289**
46	PC aa C40:3	7	0.0797	0.2527	0.1383	0.0421	0.0018	**0.0359**
53	lysoPC a C26:0	6	0.0167	0.2324	0.1711	0.0433	0.0002	**0.0165**
61	PC aa C40:2	5	0.1029	0.2888	0.1480	0.0449	0.0017	**0.0359**
73	lysoPC a C28:0	5	0.0001	0.1916	0.1741	0.0426	0.0001	**0.0165**
98	lysoPC a C26:1	3	0.0000	0.1910	0.1457	0.0458	0.0024	**0.0359**
		(B) Parameters of the Network Analysis			Associations with BMI			
Rank	Metabolite	Degree	Betweenness Centrality	Closeness Centrality	β-Coefficient	Standard Error	*p* Value	FDR-p
1	Leu	4	0.6667	1.0000	0.0053	0.0496	0.9152	0.9869
2	PC aa C38:5	4	0.6500	0.8333	0.0930	0.0470	0.0530	0.2499
3	lysoPC a C26:0	4	0.5833	1.0000	0.1711	0.0433	0.0002	0.0165
4	PC ae C40:3	4	0.5556	0.5625	0.0746	0.0474	0.1210	0.3539
5	C14:1	4	0.3333	1.0000	0.0009	0.0490	0.9860	0.9927
6	PC ae C40:4	4	0.1667	0.4737	0.0928	0.0474	0.0550	0.2499
**7**	PC ae C40:5	4	0.1667	0.4737	0.0407	0.0473	0.3928	0.6855
8	PC aa C36:3	3	0.6667	1.0000	0.1129	0.0476	0.0213	0.1606
9	PC ae C38:5	3	0.6000	0.7143	0.0490	0.0468	0.2993	0.5946
10	PC ae C38:6	3	0.6000	0.7143	0.0316	0.0481	0.5146	0.7619
15	lysoPC a C28:0	3	0.0833	0.8000	0.1741	0.0426	0.0001	**0.0165**
19	PC aa C36:4	2	1.0000	1.0000	0.1321	0.0421	0.0027	**0.0370**
30	PC aa C24:0	2	0.0000	0.6667	0.1585	0.0433	0.0006	**0.0278**
54	lysoPC a C20:4	1	0.0000	0.6667	0.1507	0.0424	0.0008	**0.0289**
57	lysoPC a C26:1	1	0.0000	0.5714	0.1457	0.0458	0.0024	**0.0359**

Rank ordered by degree. Abbreviations: lysoPC = lyso-phosphatidylcholine; a = acyl; aa = diacyl; ae = acyl-alkyl; PC = phosphatidylcholine; SM = sphingomyelin; C = acylcarnitines.

## Data Availability

Data cannot be shared publicly because they include potentially identifying and sensitive patient information.

## Supplementary Materials

The following supporting information can be downloaded at: https://www.mdpi.com/article/10.3390/metabo15020088/s1, Figure S1: Ward hierarchical clustering of a matrix of chord distance among subjects based on metabolites data; Figure S2: Bar-plot showing the average silhouette widths for k = 2 to 64 groups; Figure S3: Silhouette plot of the two-group partition from Ward clustering; Figure S4: Silhouette plot of the two-group partition from k-means partitioning.
