# Supplementary material for: Clustering-Based Identification of BMI-Associated Metabolites with Mechanistic Insights from Network Analysis in Korean Men
Source: Metabolites. 2025 Feb 2;15(2):88. doi: 10.3390/metabo15020088 (PMC11857321; doi:10.3390/metabo15020088)
Supplement: Supplementary file 1 [file metabolites-15-00088-s001.zip › metabolites-3442234-supplementary.pdf]

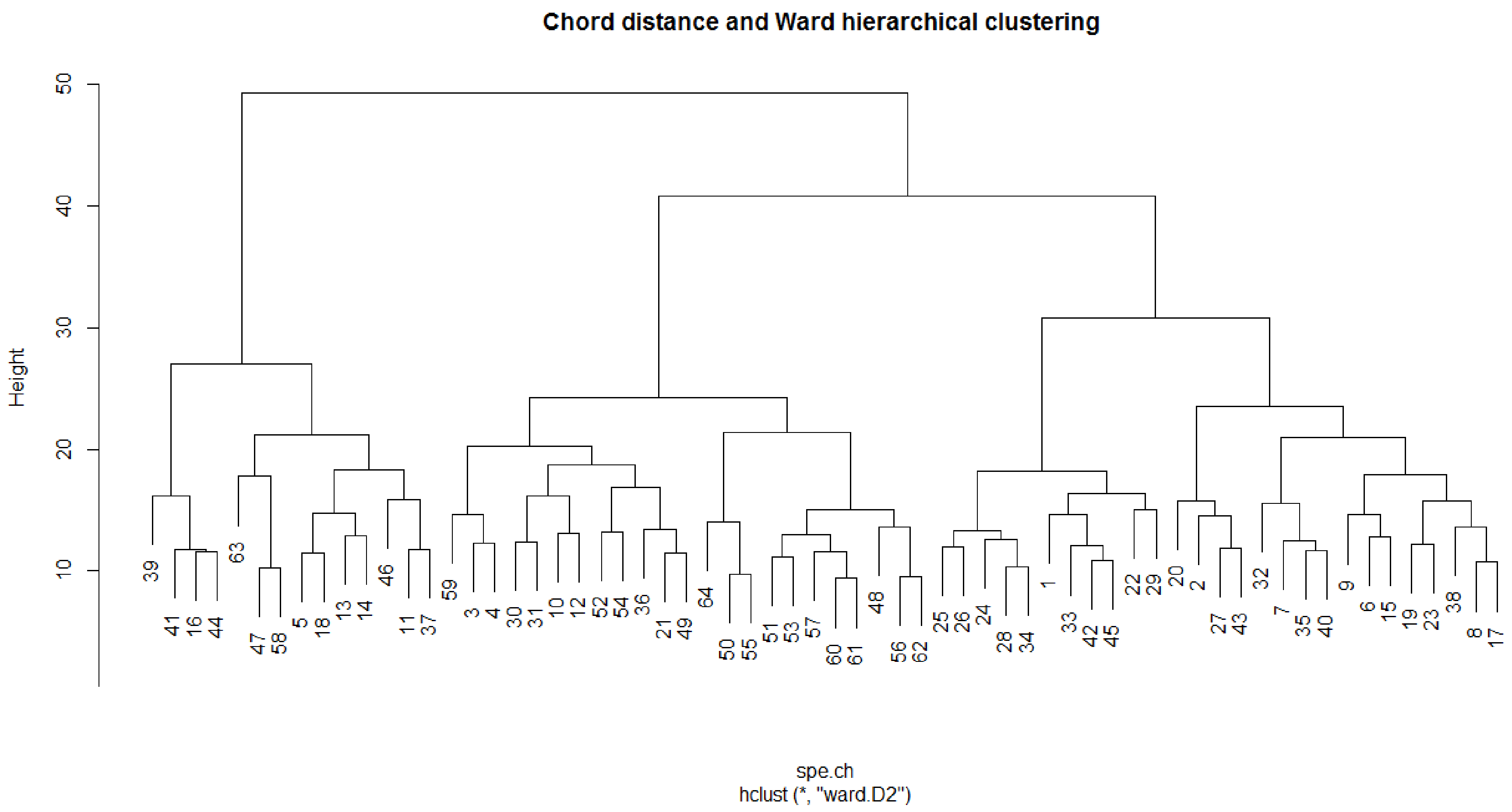


Figure S1. Ward hierarchical clustering of a matrix of chord distance among subjects based on metabolites data

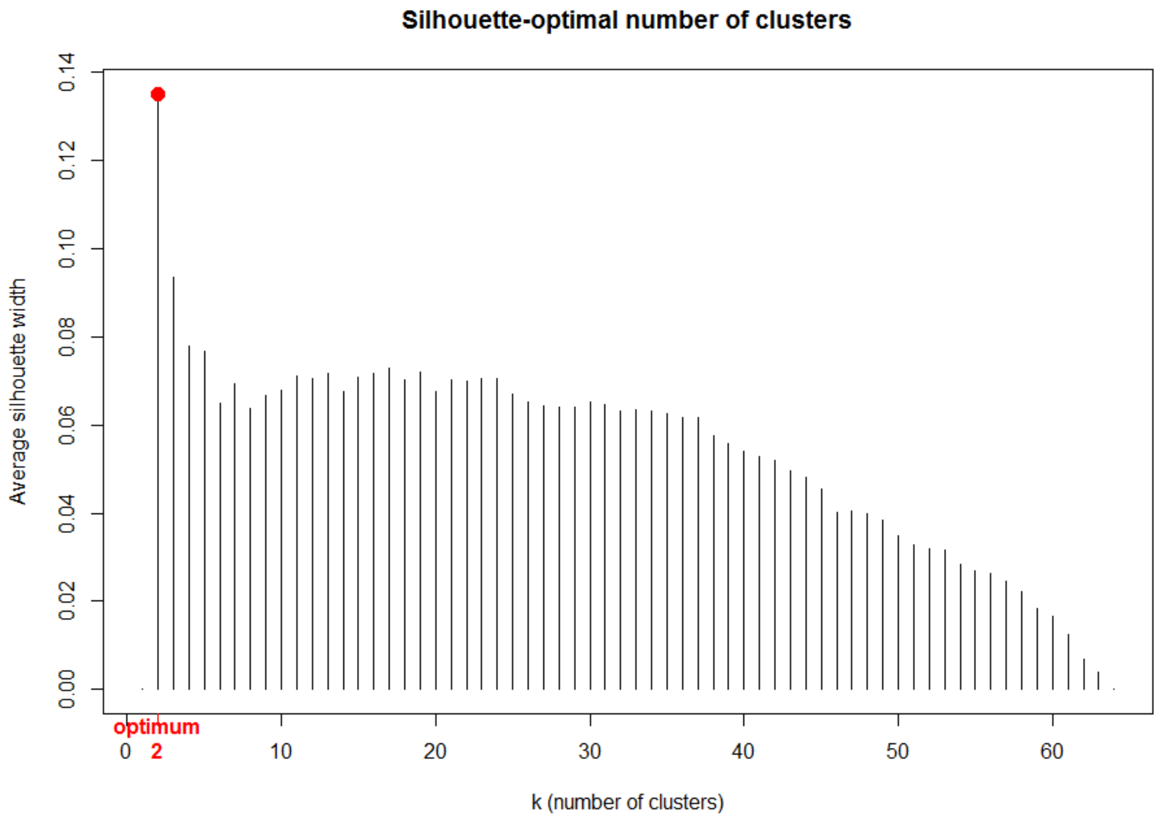


Figure S2. Bar-plot showing the average silhouette widths for k = 2 to 64 groups.

The best partition by this criterion is the one with the largest average silhouette width, i.e., in two groups.

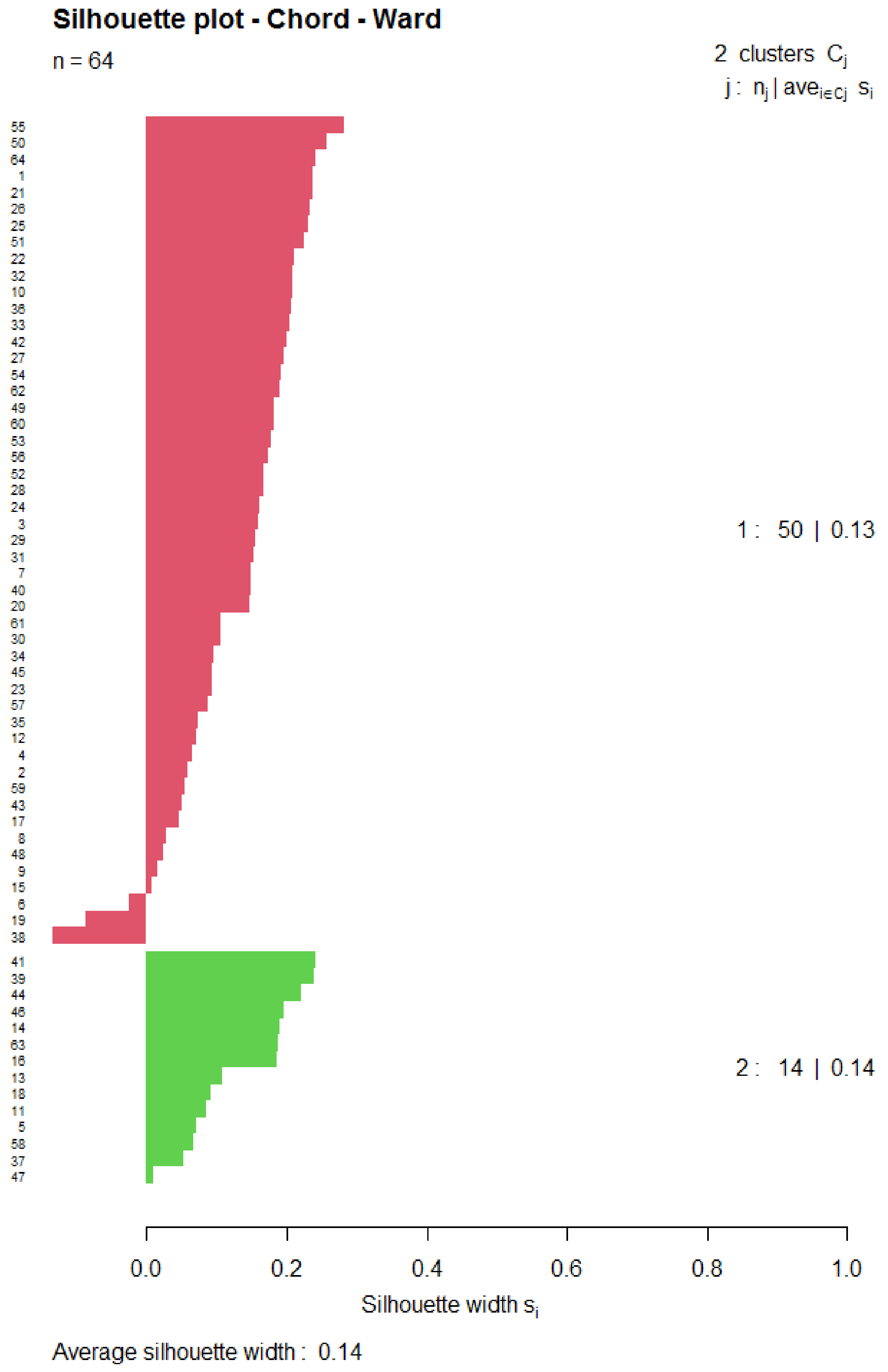


Figure S3. Silhouette plot of the two-group partition from Ward clustering

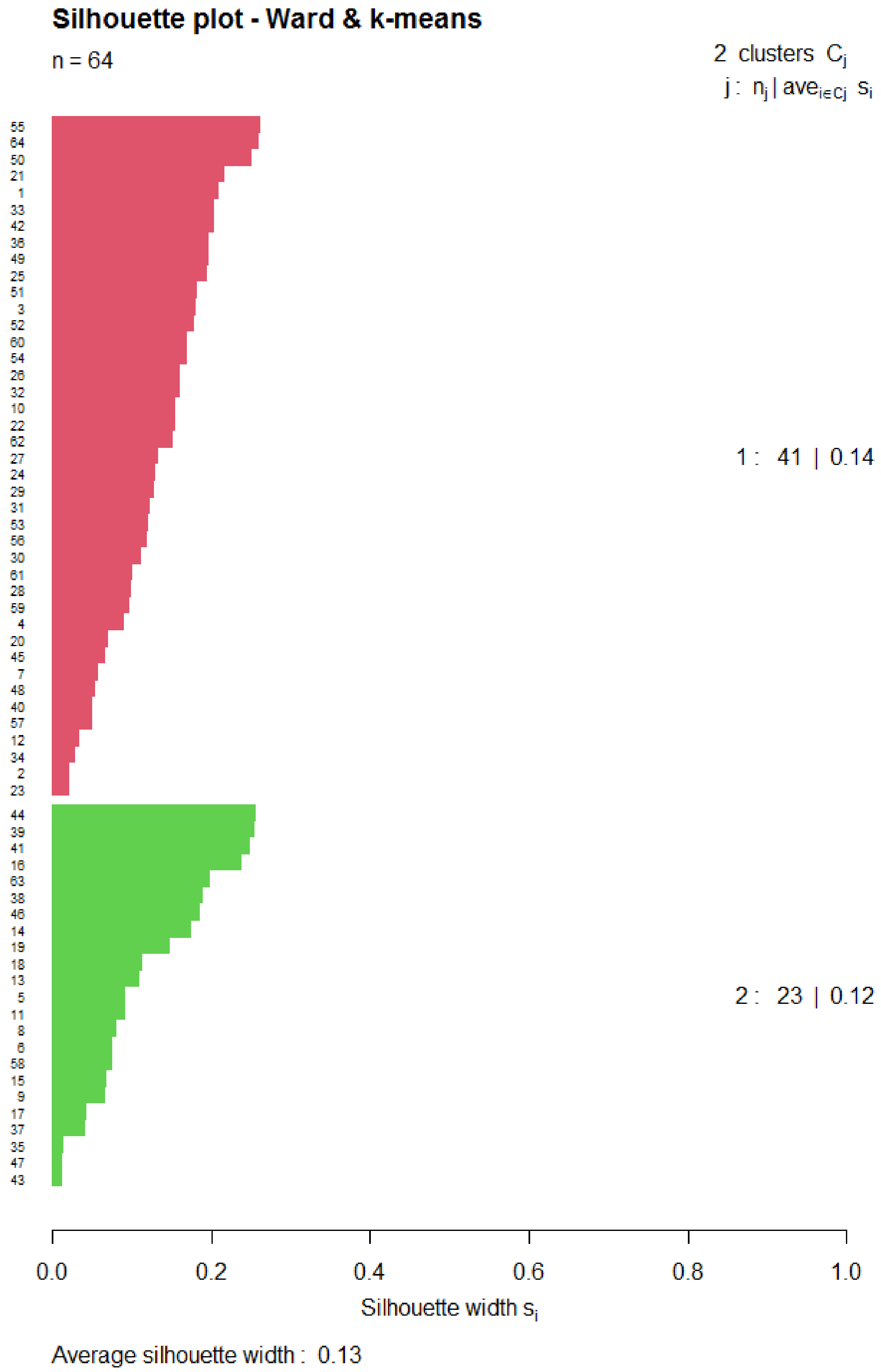


Figure S4. Silhouette plot of the two-group partition from *k*-means partitioning
